# Multiple Factors Drive Replicating Strand Composition Bias in Bacterial Genomes

**DOI:** 10.3390/ijms160923111

**Published:** 2015-09-23

**Authors:** Hai-Long Zhao, Zhong-Kui Xia, Fa-Zhan Zhang, Yuan-Nong Ye, Feng-Biao Guo

**Affiliations:** 1 Center of Bioinformatics, Key Laboratory for NeuroInformation of the Ministry of Education, School of Life Science and Technology, University of Electronic Science and Technology of China, Chengdu 610054, China; E-Mails: biohellen@gmail.com (H.-L.Z.); xiazhk@163.com (Z.-K.X.); fzzhang@cefg.cn (F.-Z.Z.); 2 Center for Information in BioMedicine, University of Electronic Science and Technology of China, Chengdu 610054, China

**Keywords:** strand composition bias, multiple factors, gene density, genomic features, COG functional category, obligate intracellular bacteria

## Abstract

Composition bias from Chargaff’s second parity rule (PR2) has long been found in sequenced genomes, and is believed to relate strongly with the replication process in microbial genomes. However, some disagreement on the underlying reason for strand composition bias remains. We performed an integrative analysis of various genomic features that might influence composition bias using a large-scale dataset of 1111 genomes. Our results indicate (1) the bias was stronger in obligate intracellular bacteria than in other free-living species (*p*-value = 0.0305); (2) *Fusobacteria* and *Firmicutes* had the highest average bias among the 24 microbial phyla analyzed; (3) the strength of selected codon usage bias and generation times were not observably related to strand composition bias (*p*-value = 0.3247); (4) significant negative relationships were found between GC content, genome size, rearrangement frequency, Clusters of Orthologous Groups (COG) functional subcategories A, C, I, Q, and composition bias (*p*-values < 1.0 × 10^−8^); (5) gene density and COG functional subcategories D, F, J, L, and V were positively related with composition bias (*p*-value < 2.2 × 10^−16^); and (6) gene density made the most important contribution to composition bias, indicating transcriptional bias was associated strongly with strand composition bias. Therefore, strand composition bias was found to be influenced by multiple factors with varying weights.

## 1. Introduction

The DNA replication process produces two identical DNA molecules from one original DNA molecule. The leading strand is synthesized continuously in the same direction as the growing replication fork and the lagging strand is replicated by the synthesis of short and separated Okazaki fragments that are then joined together to form an integrated strand [1]. According to Chargaff’s second parity rule (PR2), a single DNA strand globally has an equal percentage of base pairs (A ≈ T and G ≈ C) when there is no strand bias caused by mutation or selection [2]. After PR2 bias caused by mutation was found between the leading and lagging strands in the echinoderm and vertebrate mitochondria genomes [3], the same phenomenon has been found in an increasing number of genomes [4,5,6,7,8,9,10,11]. These biases consistently showed that the leading strand had more G than C and, to a lesser extent more T than A, while in lagging strand the bias was in the opposite direction [9,12,13].

Many researchers found that the strand bias was related to the replication process, because the accumulation of base mutations were caused by the asymmetric replication mechanism between the two strands [1,2,6,14,15]. The rule of Watson–Crick base pairing would protect cytosine from being deaminized in double-stranded DNA [16,17]. However, DNA must be separated into two single strands temporarily during replication. In single-stranded DNA, cytosine would be easier to undergo deamination and transform to thymine, which contributes towards the composition bias in genomes [16]. Researchers have found that other factors may lead to asymmetry of DNA, such as thymine dimers [18], nonsense mutations [11,16], two-fold degenerated sites of cytosine [13,19], and nucleotide usage in twofold as well as fourfold degenerate sites from third codon positions [20]. Other researchers suggested that the strand composition bias was associated with the transcription process [21,22]. The mutation and repair frequencies between coding and non-coding regions of genomes are different, and most genes are located on the leading strands [1,23]. Hence, considering the gene orientation bias, the transcription process also could induce composition bias between two replicating strands.

Thus, the mechanisms underlying nucleotide composition bias are still open to debate. In this work, we selected 1111 microbial genomes to study a number of factors that may affect strand composition bias, using a quantitative analysis approach.

## 2. Results and Discussion

### 2.1. Composition Bias in Obligate Intracellular Bacteria

Extremely strong strand composition bias has been reported in 11 bacteria, among which seven are obligate intracellular parasites [8]. The strong bias means that genes have significantly different base and codon usages between the two replicating strands [24,25,26]. Obligate intracellular bacteria live permanently in their hosts, which helps to protect them against some DNA damage [7]. Thus, during their long-term evolution, some DNA repair genes would have been lost and mutations would have accumulated, resulting in the strand composition bias that has been reported.

In this work, we analyzed the composition bias in obligate intracellular bacteria using a broader range of genomes than has been used previously. Among the 1111 genomes that we downloaded from the NCBI FTP site (see Section 3.1 for details), 83 bacteria were confirmed as obligate intracellular. The species names and access numbers are displayed in Table S1. The average *Score_composition bias_* (see Section 3.2 for details) of the 83 obligate intracellular bacteria (0.0433) was significantly higher than that of the other bacteria (0.0362) (*t*-test, *p*-value = 0.0305), and 40 of the 83 genomes were among the top scoring 258 genomes (top quarter). However, the top 10 genomes were not from obligate intracellular bacteria. Thus, the *Score_composition bias_* of obligate intracellular bacteria was stronger on the whole than that of the other species, but not always strong for an individual genome.

### 2.2. Composition Bias in Different Bacterial Phyla

**Figure 1:**
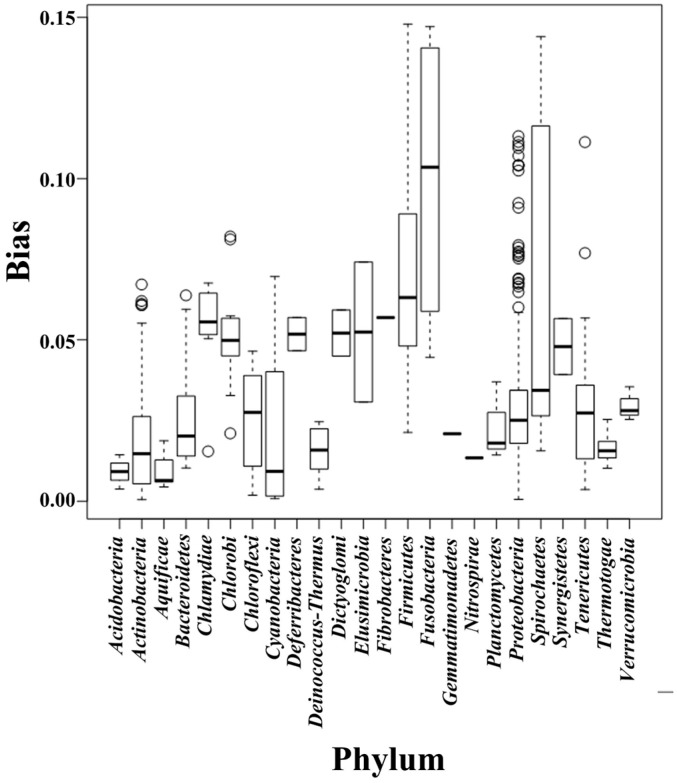
Box-and-whiskers represent for composition bias of all genomes, which sorted into 24 phyla. The bottom and top of box mark the first and third quartiles, and the band inside the box denotes the median. The ends of the whiskers in each plot represent the lowest datum still within 1.5 IQR (interquartile range) of the lower quartiles, and the highest datum still within 1.5 IQR of the upper quartiles. Any data not included between the whiskers is plotted as an outlier with a small circle. This boxplot graphically depict the different bias distribution in respective phylum.

**Figure 2:**
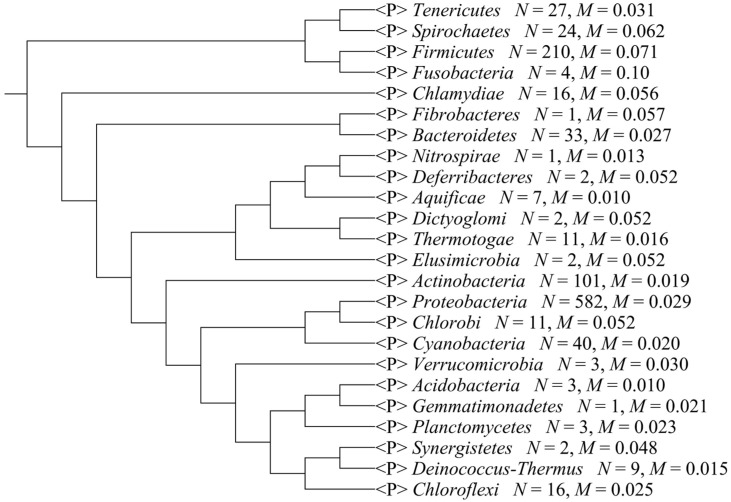
The phylogenetic tree of the 24 phyla. *N* means the total strains in a phylum, *M* means the average *Score_composition bias_* in a phylum.

We separated the 1111 microbial genomes into 24 phyla and plotted the *Score_composition bias_* for each phylum (Figure 1); the variance, standard deviation, and average *Score_composition bias_* are given in Table 1. *Fusobacteria* had the highest average *Score_composition bias_*. They are obligately anaerobic non-spore-forming Gram-negative bacteria [27]. *Firmicutes* had the second highest average *Score_composition bias_*, which is in accord with a previous study that found that strand-biased gene distribution was stronger in Firmicutes than in other bacteria [28]. To explore other features that may affect composition bias at the phylum level, we compared the size, GC content, and rearrangement frequencies of the Fusobacteria and Firmicutes genomes and found that these three features were smaller than the average values for all the other bacterial genomes; however, the gene densities in these two phyla were larger than the average values for all the other bacteria (Table 2). We reconstructed the phylogenetic tree of the 24 phyla (Figure 2) and found that the Fusobacteria and Firmicutes phyla had the closest relationship. Meanwhile, they had the top two *Score_composition bias_* (0.100 and 0.071). We also found that several other clades with close relationship had similar *Score_composition bias_*, such as among *Gemmatimonadetes*, *Planctomycetes* and *Acidobacteria*. This suggests phylogenetic relationship is one of the determinant factors of strand composition bias in bacterial genomes.

**Table 1:** Strand composition bias for each phylum ^a^.

Phylum	Standard Deviation	Variance	Mean
*Acidobacteria*	0.005309	2.82 × 10^−5^	0.009124
*Actinobacteria*	0.015749	0.000248	0.018728
*Aquificae*	0.005263	2.77 × 10^−5^	0.00957
*Bacteroidetes*	0.016805	0.000282	0.027048
*Chlamydiae*	0.012521	0.000157	0.055526
*Chlorobi*	0.018046	0.000326	0.051947
*Chloroflexi*	0.015056	0.000227	0.024993
*Cyanobacteria*	0.021638	0.000468	0.019847
*Deferribacteres*	0.007318	5.36 × 10^−5^	0.051752
*Deinococcus-Thermus*	0.007668	5.88 × 10^−5^	0.015442
*Dictyoglomi*	0.010132	0.000103	0.052093
*Elusimicrobia*	0.030697	0.000942	0.052418
*Fibrobacteres*	NA	NA	0.056901
*Firmicutes*	0.028571	0.000816	0.071236
*Fusobacteria*	0.048886	0.00239	0.099682
*Gemmatimonadetes*	NA	NA	0.020857
*Nitrospirae*	NA	NA	0.013445
*Planctomycetes*	0.012161	0.000148	0.023082
*Proteobacteria*	0.017163	0.000295	0.028607
*Spirochaetes*	0.046978	0.002207	0.062153
*Synergistetes*	0.012306	0.000151	0.047907
*Tenericutes*	0.023255	0.000541	0.030599
*Thermotogae*	0.004126	1.70 × 10^−5^	0.016197
*Verrucomicrobia*	0.005228	2.73 × 10^−5^	0.029585

^a^ All genomes are grouped by phylum, NA refer to that there is only one species in this phylum. The phylum Fusobacteria owned the highest mean bias value, and the Firmicutes comes second.

**Table 2:** Mean value of various biological characters for each phylum ^a^.

Phylum	Genome Size	GC Content	Gene Density	*gcRF*	*taRF*
*Acidobacteria*	6,581,121.33	0.602611	0.524179	0.546299	0.239179
*Actinobacteria*	4,434,386.26	0.647473	0.591745	0.655926	0.5707
*Aquificae*	1,680,594.86	0.3874153	0.514286	0.026764	0.090473
*Bacteroidetes*	3,688,038.52	0.4246355	0.553854	0.035009	0.101365
*Chlamydiae*	1,265,852.44	0.4046721	0.544713	0.022567	0.081014
*Chlorobi*	2,618,734.27	0.5079388	0.583907	0.061015	0.114787
*Chloroflexi*	2,435,937.54	0.5531583	0.519221	0.044977	0.063278
*Cyanobacteria*	3,397,176.98	0.4460103	0.508569	−0.33356	−0.55797
*Deferribacteres*	2,728,233	0.3682745	0.642415	0.012609	0.057666
*Deinococcus-Thermus*	2,411,100.11	0.66285	0.517812	−0.10793	−0.12243
*Dictyoglomi*	1,907,773.5	0.3384917	0.681195	0.01941	0.055101
*Elusimicrobia*	1,384,709.5	0.3757977	0.726988	0.014904	0.078649
*Fibrobacteres*	3,842,635	0.4805184	0.580603	0.047916	0.088216
*Firmicutes*	3,077,249.49	0.3853	0.786812	0.020021	0.081354
*Fusobacteria*	2,680,383	0.29141	0.72341	0.01046	0.05595
*Gemmatimonadetes*	4,636,964	0.6427436	0.566455	0.043068	0.055612
*Nitrospirae*	2,003,803	0.341289	0.552386	0.019141	0.07548
*Planctomycetes*	6,254,950	0.5550987	0.502151	0.116125	0.138471
*Proteobacteria*	3,506,416.55	0.5337785	0.569934	0.067462	0.135439
*Spirochaetes*	1,702,653.17	0.3721947	0.600467	0.021591	0.121083
*Synergistetes*	1,914,533	0.5454971	0.75006	0.023406	0.050368
*Tenericutes*	892,007.889	0.2794737	0.665323	−0.02018	−0.08702
*Thermotogae*	1,976,742.36	0.4028872	0.54724	0.024232	0.083806
*Verrucomicrobia*	3,998,507	0.5480856	0.51413	0.093882	0.10771
*Mean*	3,329,265.48	0.4952767	0.612158	0.092191	0.127667

^a^ Genome size, GC content and rearrangement frequency of *Fusobacteria* and *Firmicutes* are all smaller than average of each trait for all genomes, but the opposite was true for the gene density.

### 2.3. Composition Bias in Genomes with Different S Values

Selection and mutation are two primary factors that generate bias in species’ genomes during evolution. These two factors may generate biases that partially counteract each other. An *S* value can be used to measure the strength of codon usage bias as an indicator of selection bias [29]. Replicating strand composition bias can be considered to represent mutation bias. Thus, we used the *S* values for 80 bacterial genomes that were reported by Sharp *et al.* [29] to study the correlation between them and the *Score_composition bias_* of the same 80 genomes. We found that there was no significant correlation between them (Spearman’s correlation, ρ = −0.08604675, *p*-value = 0.3247). Hence, we suggest that selection and mutation may influence genome bias by different mechanisms; therefore, codon usage bias may counteract strand composition bias.

### 2.4. Composition Bias in Genomes with Different Generation Times

Microbial generation times range from a few minutes to several weeks and are affected by evolutionary factors such as environment stability, nutrient availability, and community diversity. Vieira-Silva and Rocha found that codon usage bias was correlated with growth rates [30]. Hence, we explored the relationship of generation time and *Score_composition bias_*. The bacterial generation time data were extracted from of the paper by Vieira-Silva and Rocha [30]. Our result indicated that generation time also was not significantly related with *Score_composition bias_* (Spearman’s correlation, ρ = −0.1457365, *p*-value = 0.1021). That may be the same as the reason mentioned on the *S* value.

### 2.5. Composition Bias in Genomes with Different Genome Sizes

The average sizes of the genomes in the Fusobacteria and Firmicutes phyla are smaller than average sizes of the genomes in all the bacterial phyla examined. We found that a significantly negative correlation existed between genome size and *Score_composition bias_* (Spearman’s correlation, ρ = −0.2508015, *p*-value < 2.2 × 10^−16^). This finding is similar to the results of Guo and Ning [7] who found that the genome sizes of 11 bacteria with extremely strong strand composition biases were all smaller than 2000 kb. Guo and Ning speculated that the repair mechanism might be inefficient in small bacterial genomes that had undergone reductive evolution [7]. Additionally, mutation pressure may be insufficient to surpass translational selection in larger genomes.

### 2.6. Composition Bias in Genomes with Different Gene Densities of the Leading Strand

With the availability of a large number of complete genome sequences, it has become increasing clear that the unequal distribution of genes between leading and lagging strands varies widely among different species. Numerous studies have shown that genes are generally preferentially located on the leading strand [31,32,33,34], which may be explained by the polymerase collision avoidance model [1].

We calculated the density of leading strand genes for all 1111 genomes. Our correlation analysis showed that gene density was highly positively correlated with *Score_composition bias_* (Spearman’s correlation, ρ = 0.6273871, *p*-value < 2.2 × 10^−16^). This result could be caused by DNA replication-associated mutation bias during the transcription process in which DNA decomposes into single strands. However, the DNA mutation or repair rates were quite different between transcribed and non-transcribed strands. Because most protein-coding genes are located on the leading strand, the two replication strands can have extremely different compositions [21]. Thus, the asymmetric transcription process is likely to have a major impact on the composition bias between the two replication strands.

### 2.7. Composition Bias in Genomes with Different GC Contents

GC content is the percentage of guanine and cytosine base pairs in a DNA sequence. The GC content of bacterial genomes ranges from about 20% to 70% [35]. We investigated the correlation between GC content and *Score_composition bias_* and found that a significantly negative correlation existed between them (Spearman’s correlation, ρ = −0.5026315, *p*-value < 2.2 × 10^−16^). It may be explained that genomes with high GC content will generate fewer mutations than those with low GC content [36]. However, this would inspire us that the replicating strand composition bias is caused by a complex set of factors.

### 2.8. Composition Bias in Genomes with Different Recombination Rates

Chromosomal recombination occurs as a result of deletions, duplications, inversions, and translocations in native chromosomes. Rocha [1] has shown that the recombination rate is related to strand composition bias, and has suggested that codon usage separation may be caused by low recombination rates in some obligate intracellular parasites. Wei and Guo confirm this suggestion in 11 obligate intracellular bacteria with strong strand composition bias using the *Z*-curve method [24].

Here, we explored this issue in the 1111 genomes. The recombination rates (*taRF*, *gcRF*) of each genome were calculated as described in Section 3.3. Then, the correlations between *Score_composition bias_* and both *taRF* and *gcRF* were estimated for all the genomes. We found that *taRF* and *gcRF* were both negatively associated with *Score_composition bias_* (Spearman’s correlations, ρ*_gcRF_* = −0.3746862, ρ*_taRF_* = −0.2916134, both *p*-values < 2.2 × 10^−16^).

Rocha suggested that frequent chromosomal recombination would reduce strand composition bias [1]. The base distribution in any one strand is accordant; that is, if G > C in a particular region, then a similar base distribution also will be found in other regions of the same chromosome. However, recombination would break the accordance and reduce strand composition bias.

### 2.9. Composition Bias in Different COG Functional Categories

To determine whether gene function has an impact on strand composition bias, we explored the relationship between Clusters of Orthologous Groups (COG) functional categories and composition bias for the first time.

#### 2.9.1. Percentage of Gene Number for Each COG Functional Subcategory

To explore the influence of each COG subcategory on composition bias, the correlation between the percentage of each COG functional subcategory (pCOGi; see Section 3.4 for details) and the corresponding *Score_composition bias_* was analyzed for each genome. The results, summarized in Table 3, were considered as statistically significant if the *p*-value was <1.0 × 10^−8^. Based on this cutoff value, the pCOGs of the A, C, I, and Q subcategories were negatively related to *Score_composition bias_*, and the D, F, J, L, and V subcategories showed positive correlations to *Score_composition bias_*.

Klasson and Andersson have studied gene function and composition bias [37]. They found that strong asymmetric mutation bias in endosymbiont genomes caused them to lack replication restart genes (subcategory L). Guo and Ning reported that genes associated with replication initiation and re-initiation such as *mutH*, *dnaT* and *fis* were absent in 11 obligate intracellular bacteria genomes with extreme strand composition bias [7]. However, we detected some replication initiation and re-initiation genes based on our analysis of the 1111 genomes, which indicated that COG subcategory L and composition bias was positively correlated. This is an interesting finding that we will further explore in Section 2.9.2. Rocha and Danchin [38] reported some obligate parasite bacteria with strong composition bias in which genes associated with energy metabolism were absent. This finding is mostly accord with our result that the metabolism-related genes (subcategories C, I, and Q) were all negatively correlated with composition bias, except those in subcategory F.

**Table 3:** The correlation of each Clusters of Orthologous Groups (COG) functional subcategory and strand composition bias.

COG Functional Category		*p* Value	Correlation
**Information Storage and Processing**			
J	Translation, ribosomal structure and biogenesis ^P^	8.11 × 10^−32^	0.341886
A	RNA processing and modification ^N^	2.44 × 10^−13^	−0.21728
K	Transcription	0.099239	−0.04948
L	Replication, recombination and repair ^P^	1.01 × 10^−8^	0.170797
B	Chromatin structure and dynamics	0.002404	−0.09097
**Cellular Processes and Signaling**			
D	Cell cycle control, cell division, chromosome partitioning ^P^	1.05 × 10^−45^	0.407564
Y	Nuclear structure	0.222949	0.036592
V	Defense mechanisms ^P^	3.93 × 10^−14^	0.224269
T	Signal transduction mechanisms	1.77 × 10^−7^	−0.15589
M	Cell wall/membrane/envelope biogenesis	0.609835	−0.01533
**Cellular Processes and Signaling**			
N	Cell motility	0.198305	0.038623
Z	Cytoskeleton	0.006632	−0.0814
W	Extracellular structures	0.901043	−0.00373
U	Intracellular trafficking, secretion, and vesicular transport	0.908091	0.003467
O	Posttranslational modification, protein turnover, chaperones	0.188347	−0.0395
**Metabolism**			
C	Energy production and conversion ^N^	4.51 × 10^−11^	−0.1959
G	Carbohydrate transport and metabolism	0.193919	0.039003
E	Amino acid transport and metabolism	0.417676	−0.02434
F	Nucleotide transport and metabolism ^P^	5.99 × 10^−39^	0.377498
H	Coenzyme transport and metabolism	0.01405	0.073666
I	Lipid transport and metabolism ^N^	1.22 × 10^−19^	−0.26737
P	Inorganic ion transport and metabolism	0.081681	−0.05226
Q	Secondary metabolites biosynthesis, transport and catabolism ^N^	6.65 × 10^−40^	−0.38194

N denotes significantly negative correlation between subcategories and composition bias. P denotes significantly positive correlation between subcategories and composition bias.

#### 2.9.2. Proportion of Replication and Repair Genes

The correlation between subcategory L and composition bias that we obtained is opposite to what has been found previously. To explore this result further, we collected the replication and repair genes from the KEGG pathway database and divided then into the 10 subtypes (for details see Section 3.7) based on their functions. The correlations between the percentage genes under each subtype and the *Score_composition bias_* are shown in Table 4. The gene subtypes were all positively related to composition bias, and the excision and mismatch repair subtype had the highest correlation. We suspect that genomes with strong composition bias may have generated more repair genes to balance the composition bias during evolution. However, the cause-and-effect relationship between repair genes and composition bias is not still clear; that is, which is the cause and which is the effect.

#### 2.9.3. Average Value of Times between Strong-Biased Group and Weak-Biased Group for Each Functional Subcategory

The *Diff_SBG/WBG_* (see Section 3.5 for details) for all COG subcategories is shown in Table 5. Subcategory D had the highest value (5.709 among all the subcategories, which indicated that genes involved in cell cycle control, cell division, and chromosome partitioning were present in significant numbers in the strong-biased genomes (*i.e.*, the genomes with three top 555 *Score_composition bias_* values). This result is in accordance with Lin *et al.* [39] who found that only some essential COG subcategories were situated preferentially on the leading strand and that subcategory D genes showed the most significant bias among 10 strand-biased classifications. Furthermore, both the strong-biased COG groups (SCOGs) and weak-biased COG groups (WCOGs) in all 1111 genomes were significantly related to *Score_composition bias_* (Spearman’s correlation, ρ_SCOG_ = 0.51473 and ρ_WCOG_ = −0.65945, both *p*-values < 2.2 × 10^−16^). We suggest that although the essential subcategories are similar in number in the genomes, they tend to be located on the leading strand, resulting in strong composition bias. For small genomes, the percentages of essential subcategories are higher than for large genomes, hence leading to stronger composition bias in small genomes.

**Table 4:** Average value of discrepant times (AVDT) between strong-biased group and week-biased group for each functional subcategory in descending order.

COG	AVDT	COG	AVDT
D	5.709197	C	1.086021
K	3.415376	H	1.053758
N	2.848684	F	1.046122
T	2.229241	V	1.02066
M	2.181872	E	0.99786
O	2.089135	I	0.936222
U	2.013089	P	0.914553
G	1.472415	A	0.864394
L	1.363586	Z	0.775298
B	1.266486	Q	0.64794
J	1.23429	W	0.6

**Table 5:** Relationship between each type of replication and repair genes and composition bias.

Pathway	Function	*p* Value	Correlation
ko03030	DNA replication	3.69 × 10^−10^	0.18656
ko03032	DNA replication proteins	6.70 × 10^−9^	0.172841
ko03036	Chromosome and associated proteins	3.28 × 10^−7^	0.152472
ko03400	DNA repair and recombination proteins	6.73 × 10^−10^	0.183808
ko03410	Base excision repair	2.11 × 10^−6^	0.141724
ko03420	Nucleotide excision repair	4.15 × 10^−12^	0.2059713
ko03430	Mismatch repair	9.39 × 10^−12^	0.2025802
ko03440	Homologous recombination	1.16 × 10^−10^	0.191753
ko03450	Non-homologous end-joining	0.926821	0.002759
ko03460	Fanconi anemia pathway	0.002531	0.090509

### 2.10. Conjoint Analysis of Multiple Factors and Composition Bias by Principal Component Regression

We determined the independent contribution of each genomic feature to composition bias by principal component regression. Here, we selected only the features that were significantly related with strand composition bias (*p*-values < 1.0 × 10^−8^). The replication and repair genes were not considered separately because they belong to COG subcategory L. The respective contribution is presented in detail in Table 6. The results show that among the whole contribution (*R*^2^ = 0.5104) of all the features, gene density (*R*^2^ = 0.064778) made the most contribution to strand composition bias. Thus, gene orientation bias was the primary factor that influenced base composition among the biological features tested.

**Table 6:** Principal component regression analysis of various genomic features ^a^.

Genomic Features	Genome Size	Gene Density	GC Content	*gcRF*	*taRF*	SCOGs	WCOGs	A
*R*^2^	0.0558	0.0648	0.0391	0.0004	0.0003	0.0332	0.0326	0.0122
Genomic features	C	D	F	I	J	L	Q	V
*R*^2^	0.0634	0.0348	0.0272	0.0238	0.0299	0.0371	0.0262	0.0297

^a^ Detail values for each of the genomic features and strand composition bias are listed in Table S2.

## 3. Experimental Section

### 3.1. Data Source

We retrieved 1111 bacterial genome sequences from the NCBI FTP site in September 2010. Among them, 76 bacteria had multiple strains and hence the 1111 bacteria belonged to only 703 species. We used all sequenced bacterial genomes at that time, rather than sampling the genomic data to analyze.

The origin and terminus of DNA replications were obtained from the Doric database [40] in July 2011. This information was used to separate genes onto leading and lagging strands.

The genes related to DNA repair and replications were extracted from the KEGG Pathway database [41] in April 2013.

### 3.2. Computation of Strand Composition Bias

Strand composition bias of a whole genome was obtained as:

(1) $${Score}_{Composition\;Bias}=\frac{\left|G\;-\;C|\;+\;|T\;-\;A\right|}{Chromosome\;Length}$$

where *G*, *C*, *T*, and *A* are the numbers of corresponding bases in leading strands. According to the principle of complementary base pairing, strand composition bias in lagging strands is equal to that of the leading strand.

### 3.3. Computation of Counteracting Effect of Recombination

Strand composition bias was measured by the mean value of *G* − *C* + *T* − *A*. Recombination may change the natural order of nucleotides, so to counteract some usual bias and finally lower the strength of the whole bias, we introduced two values, *gcRF* and *taRF*, to roughly reflect this effect of recombination. *gcRF* was calculated as:

(2) $$\bar{gcBias}=\frac{\sum_{i=1}^{N}\frac{G_{i}-C_{i}}{L_{i}}}{N}$$

(3) $$gcRF=\frac{\sum_{i=1}^{N}{\left(\frac{G_{i}-C_{i}}{L_{i}}-\bar{gcBias}\right)}^{2}}{(N-1)\times \bar{gcBias}}$$

and *taRF* and was calculated as:

(4) $$\bar{taBias}=\frac{\sum_{i=1}^{N}\frac{T_{i}-A_{i}}{L_{i}}}{N}$$

(5) $$taRF=\frac{\sum_{i=1}^{N}{\left(\frac{T_{i}-A_{i}}{L_{i}}-\bar{taBias}\right)}^{2}}{(N-1)\times \bar{taBias}}$$

where *G_i_*, *C_i_*, *T_i_*, and *A_i_* are the numbers of corresponding bases of the *i^th^* leading strand gene; *L_i_* is the length of the corresponding gene; and *N* is the total number of genes in the leading strand. Usually, the higher the two values are, the higher the frequency of counteracting recombination occurs.

### 3.4. Computation of the Percentage of Each COG Functional Subcategory

The percentage of each COG functional subcategory (pCOG) was calculated as:

(6) $${pCOG}_{i}=\frac{N_{COG\;i}}{N_{COG}}\;\;i=A\sim Z,\;\;\text{except}\;\;R,S,X$$

where *i* is the *i^th^* subcategory and *N_COGi_* is the number of genes with the *i^th^* subcategory in a genome. *N_COG_* is the total number of genes within all the COG subcategories.

### 3.5. Computation of Average Value of Differences between Strong-Biased Group and Weak-Biased Group for Each Functional Subcategory

We grouped the genomes with the top 555 *Score_composition bias_* values as the strong-biased group (SBG), and the remaining genomes as the weak-biased group (WBG) and count the number of genes in each COG subcategory for all the species in each group separately. For each COG, we defined an indicator, *Diff_SBG/WBG_*, to measure the differences between the two groups as:

(7) $${Diff}_{SBG/WBG}=\frac{N_{SBG}}{N_{WBG}}$$

where *N_SBG_* is the number of genes in each COG subcategory in the SBG, and *N_WBG_* is the number of genes in each COG subcategory in the WBG.

Finally, we defined another indicator, *Diff_COG_*, for each COG functional subcategory as:

(8) $${Diff}_{COG\;i}=\frac{\sum_{j=1}^{N}{Diff}_{SBG/WBG\;j}}{N}\;\;i=A\sim Z,\;\;\text{except}\;\;R,S,X$$

where *i* is the *i^th^* subcategory of the 23 COG functional subcategories; *j* is the *j^th^* gene in *i^th^* subcategory; and *N* is the total number of genes in *i^th^* subcategory.

### 3.6. Proportion of SCOGs and WCOGs

Subcategories with *Diff_COG_* > 5 were defined as strong-biased COG groups (SCOGs), and subcategories with *Diff_COG_* < 0.2 were defined as weak-biased COG groups (WCOGs). Then, the proportions of SCOGs and WCOGs in each genome were calculated.

### 3.7. Proportion of Replication and Repair Genes

We download the genes associated with replication and repair from the Kyoto Encyclopedia of Genes and Genomes (KEGG) Pathway database [41]. Ten pathways are classified under replication and repair; namely, DNA replication, DNA replication proteins, chromosome and associated proteins, DNA repair and recombination proteins, base excision repair, nucleotide excision repair, mismatch repair, homologous recombination, non-homologous end-joining, and Fanconi anemia pathway. Then, we computed the proportion of genes associated with each classification in each genome.

### 3.8. Statistical Analyses

The correlations between various genomic features and the strand composition bias were measured by Spearman’s rank correlation coefficient, which is a nonparametric measure of statistical dependence between two factors. It uses a monotonic function to assess how well the relationship between two variables. Rho of Spearman’s rank correlation is used to reflect the intensity of correlation between variables of statistical indicators and the absolute value of rho reflects the relative significance between two variables. For example, a rho value of −0.14 is less significant than a rho value of −0.25. The *p*-value of Spearman’s correlation is used for measuring significance of correlation between two variables. In this work, it is considered a significant correlation if the *p*-value <0.05. The independent contribution of each feature to the bias was confirmed statistically by principal component regression analysis. All statistical analyses were conducted using the freely available R package (https://cran.r-project.org/).

## 4. Conclusions

Strand composition bias has been reported in different genomes over many years. The bias might be driven by multiple factors. In this work, we explored the relationship between strand composition bias and various genomic features. The results show that multiple factors are related to replication strand composition bias. Together, these factors play a major role and our principal component regression analysis showed that their contribution to replication strand composition bias accounted for over 50% of the bias. Gene orientation bias had the highest independent contribution, which indicates that the transcription process is likely to have a major impact on the composition bias between two replication strands. For most of the factors, we, for the first time, quantitatively measured their contribution to strand composition bias. Thus, so far, this study is the first integrative analysis of strand composition bias in prokaryotes. The results will help understand the underlying mechanisms of how such bias is generated.

## Supplementary Materials

Supplementary materials can be found at http://www.mdpi.com/1422-0067/16/09/23111/s1.
